# Anxiolytic property of hydro-alcohol extract of *Lactuca sativa* and its effect on behavioral activities of mice

**DOI:** 10.7555/JBR.27.20120059

**Published:** 2012-12-12

**Authors:** Singapura Nagesh Harsha, Kandangath Raghavan Anilakumar

**Affiliations:** Applied Nutrition Discipline, Defence Food Research Laboratory, Mysore 570011, India

**Keywords:** *Lactuca sativa*, anxiety, nitrite, open field test (OFT), elevated plus maze test (EPM), malondialdehyde (MDA)

## Abstract

*Lactuca sativa*, belonging to the Asteraceae family, is a leafy vegetable known for its medicinal properties. This study aimed to understand the mechanism of *Lactuca sativa* extract with respect to pharmacological action.We investigated the anxiolytic effects of hydro-alcoholic extract of leaves of *Lactuca sativa* on mice. The behavioral tests performed on mice models to assess anti-anxiety properties were: open field test (OFT), elevated plus maze test (EPM), elevated T maze test, and marble burying test. Increased locomotor activity and time spent in the “open-arm” were observed in extract fed group. Malondialdehyde (MDA) and nitrite levels were decreased, catalase and glutathione levels were increased in *Lactuca sativa* treated mice. The data obtained in the present study suggests that the extract of *Lactuca sativa* can afford significant protection against anxiolytic activity.

## INTRODUCTION

Reactive oxygen species (ROS) have been implicated in many disease states ranging from arthritis and connective tissue disorders to carcinogenesis, aging, physical injury, infection and acquired immune-deficiency syndrome[1]. Because a majority of diseases/disorders are mainly linked to oxidative stress due to free radicals[2], which are fundamental to any biochemical process and represent an essential part of aerobic life and metabolism[3], it is of paramount interest to study the free radical scavenging properties of plants or plant foods. Oxidative stress has also been implicated in depression, anxiety disorders and high anxiety levels[4]. Flavonoids and phenolic compounds, which are widely distributed in plants, have been reported to exert free radical scavenging abilities, anti-inflammatory, anticarcinogenic, and anxiolytic properties[5],[6].

*Lactuca sativa*, belonging to the Asteraceae family, is an important leafy vegetable known for its medicinal properties. The whole plant has been used in the treatment of stomach problems to stimulate digestion, to enhance appetite and relieve inflammation[7] via the anti-inflammatory activities of triterpene lactones[8]. *Lactuca sativa* gives protection against *D*-galactose-induced oxidative stress and reduces accumulation of lipofuscin granules[9]. *Lactuca sativa* is known to be rich in antioxidants viz. quercetin, caffeic acid, vitamin C[10], carotenoids[11], and phytols[12]. The major components present in *Lactuca sativa* extract are 15-oxalyl and 8-sulfate conjugates of the guaianolide sesquiterpene lactones, lactucin, deoxylactucin, and lactucopicrin[13]. The antioxidant activity of *Lactuca sativa* has been reported to prevent chronic diseases related to oxidative stress such as cancer[14]. Considering the varied beneficial activities reported in the traditional system of medicine as well as the recent reports, this study aimed to study the anxiolytic effects of hydro-alcoholic extract of leaves of *Lactuca sativa* on mice.

## MATERIALS AND METHODS

### Chemicals

Thiobarbituric acid, 5, 5′-dithio-bis (2-nitrobenzoic acid), H_2_O_2_, 2,2′-azino-bis(3- thylbenzothiazoline-6-sulphonic acid), *N*-(1-naphthyl)-ethylenediamine dihydrochloride and sulfanilamide were purchased from HiMedia, Mumbai, India. All other chemicals and reagents were of analytical grade.

### Extraction

A 250 g sample of of crushed *Lactuca sativa* was used for extraction. The sample was soaked overnight in 70% alcohol (30:70) and filtered using Whatman No.1 paper. The process was repeated twice by adding fresh solvent every time. The pooled extract was subjected to flash evaporation followed by lyophilization. The lyophilized sample was further analyzed to determine its anxiolytic property.

### Animal experiment

Animal studies were conducted according to the Institute Animal Ethical Committee regulations and were approved by the Committee for the purpose of the control and supervision of experiments on animals. Male mice weighing 25–30 g were selected from the stock colony of Defence Food Research Laboratory, Mysore, India. The mice were housed in an acrylic fiber cage in a temperature-controlled room (temperature 25±2°C) and maintained in 12 hours light/dark cycle with free access to a pellet diet and drinking water.

### Experimental design

The extract of *Lactuca sativa* was suspended in a vehicle comprising of 1% Tween 20 (W/V) in distilled water. Various doses viz., 100, 200 or 400 mg/kg of 70% alcohol extract of *Lactuca sativa* were prepared by suspending the dried extracts in vehicle. Mice were randomly chosen and grouped based on their body weights. The doses were adjusted to orally administer 0.25 mL of the extract per day for 7 days. Diazepam 1 mg/kg body weight suspended in the vehicle was used as standard anxiolytic. The suspending vehicle (0.25 mL) was used as control. All the behavioral tests were performed 60 minutes after the administration of respective treatments.

### Behavioral tests

#### Elevated plus-maze test

The test procedure and scoring methodology for the elevated plus-maze test were described by Kulkarni[15]. In brief, the apparatus was composed of two open (30 cm×5 cm×0.25 cm) and two enclosed (30 cm×5 cm×15 cm) arms that radiated from a central platform (5 cm×5 cm) to form a plus sign. A slightly raised edge on the open arms (0.25 cm) provided an additional grip for the animals. The plus-maze was elevated to a height of 40 cm above the floor level by a single central support. The number of entries into and the time spent in each of the two types of arms were counted during a 5 minutes test period. The open-arm entries and open-arm time were used as indices of anxiety. A mouse was considered to have entered an arm when all four paws were on the arm.

#### Open field test

Spontaneous motor activity was evaluated in open field test according to Bhattacharya & Satyan[16]. The open field apparatus was made up of a 56 cm×56 cm black plexiglass square. The entire apparatus floor was divided into 16 squares of identical dimension. The entire room, except the open field apparatus, was kept dark during the experiment. One h after vehicle/standard/extract treatment, each animal was placed at one corner of the apparatus and the behavioral aspects were noted during the next 5 minutes.

#### Elevated T-maze test

The test procedure and scoring methodology for the elevated T-maze test were described by Viana et al.[17]. In brief, the apparatus was composed of two open (30 cm×5 cm×0.25 cm) arms and one enclosed (30 cm×5 cm×15 cm) arm that radiated from a central platform (5 cm×5 cm) to form a plus sign. A slightly raised edge on the open arms (0.25 cm) provided an additional grip for the animals. The T-maze was elevated to a height of 40 cm above the floor level by a single central support. The number of entries into, and the time spent, in each of the two types of arm, were counted during a 5 minutes test period. The open-arm entries and open-arm time were used as indices of anxiety.

#### Marble burying test

A modified procedure based on Yamada et al.[18] was employed. Mice were placed individually in plastic cages with the designated bedding material for 30 minutes (habituation period) and then placed into waiting cages. Twelve glass marbles were then evenly spaced 3 cm apart on a 4-cm layer of bedding material in the habituation cages. Mice were then reintroduced into the same cage in which they had been habituated. After 30 minutes, the marble burying period was terminated by removing the mice, and the number of marbles that were more than two-thirds covered with bedding material were counted. All behavioral recordings were carried-out using ANY MAZE software from Columbus Instruments, Ohio, USA. The animals were sacrificed after behavioral parameter testing. Blood and brain samples were collected and stored in -80°C for further biochemical analysis.

### Biochemical studies

#### Total antioxidant assay

ABTS radical scavenging assay was adopted from the method of Re et al.[19]. The ABTS assay using serum was carried-out to determine the antioxidant present.

### Estimation of nitrate, thiobarbituric acid-reactive substances (TBARS), reduced glutathione, and catalase activity

Brain tissue homogenate was used for estimation of nitrate, thiobarbituric acid-reactive substances (TBARS), catalase and glutathione[20]-[23].

#### Estimation of protein content

The protein content of brain tissue was estimated using bovine serum albumin as standard by the method of Lowry et al.[24].

### Statistical analysis

Experimental results were expressed as mean±SD. All measurements were replicated three times. The data were analyzed by an analysis of variance, i.e. one way ANOVA using Micro Cal Origin version 3.0. *P* < 0.05 was considered to be statistically significant.

## RESULTS

### Elevated plus maze test

**Fig. 1:**
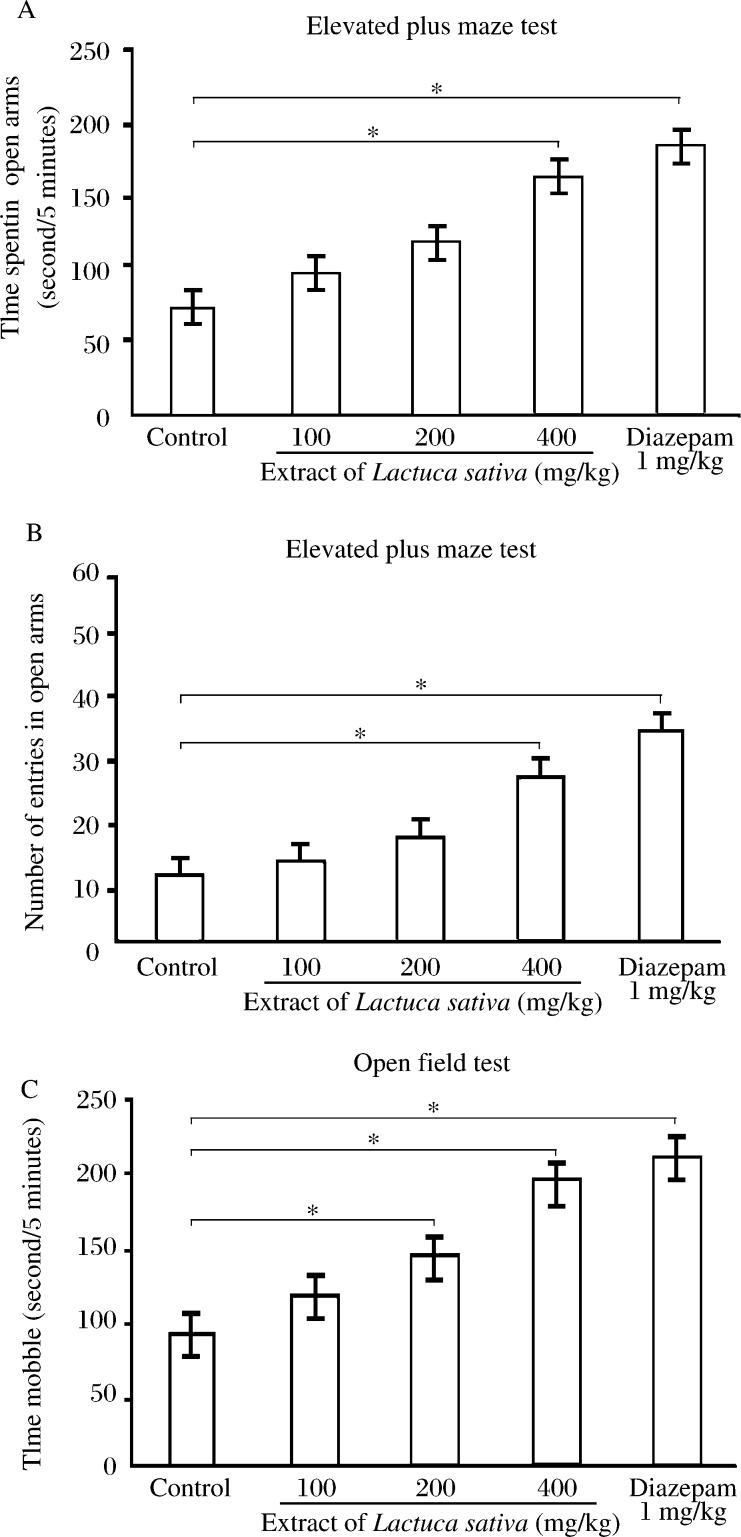
Effects of diazepam and the 70% ethanol extract of *Lactuca sativa* leaves on the time spent in the open-arms (A) and the number of entries in open-arms (B) in the elevated plus-maze and time mobile in the open field test (C) for a duration of 5 minutes each on mice. The plant extract, diazepam or control was administered 60 minutes prior to test. Data are presented as mean values (±SD), *n* = 6, *P < 0.05.

The yield of the *Lactuca sativa* extract was 70.2 mg/g of dry powder. Drugs that increase open-arm exploration are considered anxiolytic and the reverse holds true for anxiogenics. In our study, we observed that extract at doses of 200 and 400 mg/kg (*P* < 0.001) significantly increased the number of entries and time spent in the open-arms, with associated decrease in closed-arms when compared to the control treated group (***Fig. 1***). Plant extract at 100 mg/kg had no significant effects on any of the parameters that were measured on the elevated plus maze.

### Open field test

Anxiolytic treatment decreases anxiety-induced inhibition of exploratory behavior. Diazepam 1 mg/kg significantly (*P* < 0.001) increased ambulation, activity at the center and total locomotion. Similar results were exhibited by the extracts in the open field test. The *Lactuca sativa* extract at 200 and 400 mg/kg showed a significant (*P* < 0.001) increase in total locomotion (***Fig. 1***). However, no significant effects were produced by the administration of 100 mg/kg of the plant extract of *Lactuca sativa* leaves.

### Elevated T maze test

Animals treated with diazepam showed a significant increase in the time spent in the open-arms and decreased time spent in closed-arms, as well as an increase in the number of entries in the open-arms (***Table 1***). Animals treated with 70% ethanol extracts of *Lactuca sativa* showed an increase in time spent in open-arm and in the number of entrances into the open-arms, compared to untreated group. The dose at 400 mg/kg body weight was comparable to that of the diazepam group.

**Table 1:** Effect of 70% alcohol extract of LS on marble burying test

Group	Number of marbles buried
Control	4.50±0.90
100 mg/kg (LS)	3.25±1.20
200 mg/kg (LS)	2.50±0.50*
400 mg/kg (LS)	2.25±0.90*
1 mg/kg (diazepam)	1.75±0.90*

Data are presented as mean values (±SD), *n* = 6. *P < 0.05 compared with vehicle-treated control. LS: *Lactuca sativa.*

### Marble burying test

Extract treatment at the dosage levels of 200 and 400 mg/kg body weight resulted in decrease in the number of marbles buried as compared to controls (***Table 2***).

**Table 2:** Effect of 70% alcohol extract of *Lactuca sativa* on elevated T maze test

Group	Time in open arm (s/5 minutes)	Time mobile in open arm (s/5 minutes)	Number of entries to open arm
Control	53.68±9.60	42.67±11.80	9.75±5.10
100 mg/kg (LS)	87.32±10.30	46.25±9.70	18.50±4.60
200 mg/kg (LS)	99.80±10.07*	77.72±13.00*	22.00±6.30*
400 mg/kg (LS)	170.88±12.90**	99.82±15.00*	32.50±8.50*
1 mg/kg (diazepam)	192.97±9.70**	131.45±10.00**	40.50±7.50**

Data are presented as mean values (±SD), *n* = 6. *P < 0.05; **P < 0.01 compared with vehicle-treated control. LS: *Lactuca sativa*.

### Total antioxidant assay

**Fig. 2:**
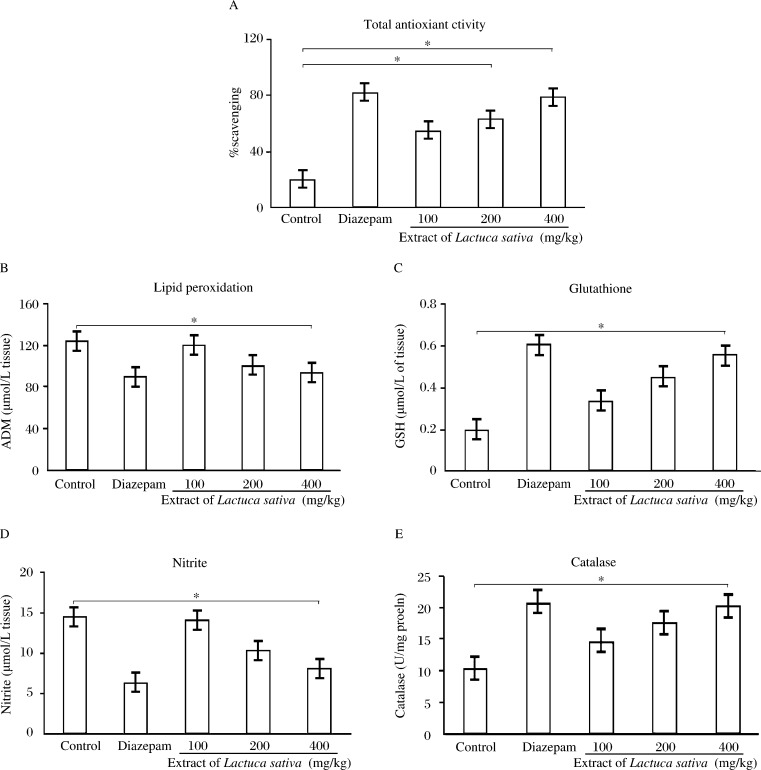
Effect of 70% ethanol extract of *Lactuca sativa* leaves on total antioxidant activity in serum (A), and malondialdehyde (B), glutathione (GSH) (C), nitrite levels (D) and catalase activity (E) of brain in mice. Values expressed as mean±SD, *n* = 6. *P < 0.05 compared with the control group.

In comparison to the control group, there was a significant increase in the total antioxidant level in serum samples from the diazepam and plant extract treated groups (***Fig. 2***).

### Thiobarbituric acid reactive substances

A significant increase in the TBARS level in the brain was observed with the control groups. Treatment with drugs and plant extract decreased the level of TBARS levels in the brain and liver samples. Among the treatments, *Lactuca sativa* administered at 200 and 400 mg/kg body weight and diazepam produced better response, indicating a protective effect (***Fig. 2***).

### Nitrites

A significant increase in the brain nitrite level was observed with the control group. Administration of diazepam and plant extract decreased brain nitrite levels. Among the treatments, *Lactuca sativa* extract 400 mg/kg body weight and diazepam produced better response, indicating a protective effect on oxidative stress (***Fig. 2***).

### Catalase

Significant decrease in the catalase level was observed in the control group. Administration of *Lactuca sativa* 400 mg/kg body weight reversed the reduction of catalase. Diazepam significantly attenuated the oxidative effect in brain (***Fig. 2***).

### Total glutathione

In the control group, a significant decrease in glutathione level was observed. Treatment of diazepam and plant extract increased the level of glutathione in brain (***Fig. 2***).

## DISCUSSION

Several plants that are used in folk medicine to diminish anxiety are reported to bring about an increase in the exploration of the open-arms in the elevated plus maze test[25]. In elevated plus maze tests, mice will normally prefer to spend much of their allotted time in the closed-arms. This preference appears to reflect an aversion towards open-arms that is generated by fear of open spaces. The animals also exhibited aggressive behavior and fighting behavior was also observed in some animals. These results clearly show that glutamate in rats can lead to excitation and oxidative stress resulting in neurodegeneration[26]. The locomotor activity of the mice was analyzed using the open field test. Elevated T maze pharmacological studies provide evidence that the open-arm avoidance and escape behaviors are expressions of emotional states akin to the generalized anxiety and panic disorder, respectively[27]. The marble burying test is a useful model of neophobia[28] and obsessive-compulsive behavior[29]. Oxidative stress may result from overproduction of H_2_O_2_ and resultant reactive oxygen species, and/or decreased efficiency of inhibitory and free radical scavenging systems[30]. Increased levels of NO result in the formation of long-lived oxidant species, such as peroxinitrite, and culminate in local neural damage[31]. Catalase scavenges H_2_O_2_; catalase in the presence of Fe may lead to production of hydroxyl radical. These ROS along with singlet molecular oxygen may attack lipids proteins and DNA of cells resulting in increased oxidative stress. Glutathione redox function is used to remove toxic peroxides formed in the normal course of growth and metabolism under aerobic conditions. In our earlier work the HPLC analysis of the polyphenols of LS revealed the presence chlorogenic acid, vanillin, epicatechin, caffeic acid, rutin hydrate, sinapic acid, quercetin-3-rhamnoside, p-coumeric acid and quercitin. The presence of these components in the extract may possess anxiolytic activity.

On the basis of the results obtained in the present study, it is concluded that the ethanol extract of *Lactuca sativa* exhibits potent anxiolytic property. These in vivo assays indicate that this plant extract is a significant source of natural antioxidant which has a capacity to decrease anxiety, which might be helpful in preventing the progression of anxiety. However, the components responsible for the antianxiety activity are currently unclear.
